# A Comparison Between 12 Versus 20 Weeks of Trimethoprim-sulfamethoxazole as Oral Eradication Treatment for Melioidosis: An Open-label, Pragmatic, Multicenter, Non-inferiority, Randomized Controlled Trial

**DOI:** 10.1093/cid/ciaa1084

**Published:** 2020-07-29

**Authors:** Siriluck Anunnatsiri, Wipada Chaowagul, Prapit Teparrukkul, Ploenchan Chetchotisakd, Kittisak Tanwisaid, Supphachoke Khemla, Surapong Narenpitak, Moragot Pattarapongsin, Wirod Kongsawasd, Pornrith Pisuttimarn, Wilawan Thipmontree, Piroon Mootsikapun, Seksan Chaisuksant, Wirongrong Chierakul, Nicholas P J Day, Direk Limmathurotsakul

**Affiliations:** 1 Faculty of Medicine, Khon Kaen University, Khon Kaen, Thailand; 2 Melioidosis Research Centre, Khon Kaen University, Khon Kaen, Thailand; 3 Sunpasitthiprasong Hospital, Ubon Ratchathani, Thailand; 4 Nakhonphanom Hospital, Nakhonphanom, Thailand; 5 Udon Thani Hospital, Udon Thani, Thailand; 6 Chaiyaphum Hospital, Chaiyaphum, Thailand; 7 Srisaket Hospital, Srisaket, Thailand; 8 Khon Kaen Hospital, Khon Kaen, Thailand; 9 Maharat Nakhonratchasima Hospital, Nakhonratchasima, Thailand; 10 Faculty of Tropical Medicine, Mahidol University, Thailand; 11 Centre for Tropical Medicine, and Global Health, Nuffield Department of Medicine, University of Oxford, Oxford, United Kingdom

**Keywords:** melioidosis, eradication, treatment, duration

## Abstract

**Background:**

Treatment of melioidosis comprises intravenous drugs for at least 10 days, followed by oral trimethoprim-sulfamethoxazole (TMP-SMX) for 12 to 20 weeks. Oral TMP-SMX is recommended for 12 weeks in Australia and 20 weeks in Thailand.

**Methods:**

For this open-label, pragmatic, multicenter, noninferiority, randomized controlled trial, we enrolled patients with culture-confirmed melioidosis who had received oral eradication treatment for 12 weeks and had no clinical evidence of active melioidosis. We randomly assigned patients to stop treatment (12-week regimen) or continue treatment for another 8 weeks (20-week regimen). The primary end point was culture-confirmed recurrent melioidosis within 1 year after enrollment. The noninferiority margin was a hazard ratio (HR) of 2.0. The secondary composite end point, combining overall recurrent melioidosis and mortality, was assessed post hoc.

**Results:**

We enrolled 658 patients: 322 to the 12-week regimen and 336 to the 20-week regimen. There were 5 patients (2%) in the 12-week regimen and 2 patients (1%) in the 20-week regimen who developed culture-confirmed recurrent melioidosis (HR, 2.66; 95% confidence interval [CI], .52–13.69). The criterion for noninferiority of the primary event was not met (1-sided *P* = .37). However, all-cause mortality was significantly lower in the 12-week regimen group than in the 20-week regimen group (1 [.3%] vs 11 [3%], respectively; HR, 0.10; 95% CI, .01–.74). The criterion for noninferiority of the secondary composite end point, combining overall recurrent melioidosis and mortality, was met (1-sided *P* = .022).

**Conclusions:**

Based on the lower total mortality and noninferiority of the secondary composite end point observed, we recommend the 12-week regimen of TMP-SMX for oral eradication treatment of melioidosis.

**Clinical Trials Registration:**

NCT01420341.

## Teaser

A comparison study between 12- and 20-week regimens of trimethoprim-sulfamethoxazole (TMP-SMX) as oral eradication treatment for melioidosis shows that 12 weeks of TMP-SMX is adequate for patients who have no clinical evidence of active melioidosis after 12 weeks of treatment.

Melioidosis is a difficult-to-treat infection caused by the Gram-negative bacillus *Burkholderia pseudomallei*, found in soil and water [1]. The disease is considered highly endemic in northeast Thailand and northern Australia, where the annual incidence is up to 50 cases per 100 000 people [2, 3]. Melioidosis is also increasingly reported in tropical countries, including much of Asia, Africa, and Central and South America [4–6]. A modelling study estimated that there are about 165 000 human melioidosis cases per year, with 89 000 (54%) deaths [7]. First-line initial antimicrobial treatment is parenteral ceftazidime or a carbapenem drug for at least 10 days [1]. To prevent recurrent melioidosis, oral eradication therapy after the end of initial parenteral therapy is needed. In Thailand, recurrent melioidosis has been reported to occur in 16% of cases within 10 years of the primary infection, with a case fatality rate of 24% [8, 9]. Most recurrent cases occurring during the first year are a recrudescence or relapse of the primary infection, while most recurrences occurring after 1 year are reinfections [8, 9].

The recommended oral antimicrobial regimen is trimethoprim-sulfamethoxazole (TMP-SMX) for at least 12 to 20 weeks [1]. The 12-week TMP-SMX regimen is recommended in Australia based on multiple Australian cohort studies reporting low rates of recurrent melioidosis (2%) among those who received TMP-SMX for 3 months [10, 11]. The 20-week oral eradication treatment regimen is recommended in Thailand based on a randomized controlled trial (RCT) showing that poor compliance with the 20-week regimen was associated with a higher risk of recurrence [12]. All following RCTs conducted in Thailand used a 20-week TMP-SMX–based regimen [13–16]. The most recent RCT found that TMP-SMX was not inferior to TMP-SMX plus doxycycline, and led to TMP-SMX alone being recommended for the oral eradication treatment of melioidosis [16]. However, the discrepancy in the recommended duration of therapy has not been resolved. We proposed that TMP-SMX for 12 weeks is an adequate treatment for melioidosis, and conducted a clinical trial to compare the efficacy of 12 versus 20 weeks of TMP-SMX for the oral eradication treatment of melioidosis.

## METHODS

### Study Designs and Participants

We conducted an open-label, pragmatic, multicenter, noninferiority RCT in 8 tertiary care hospitals in northeast Thailand. We enrolled adult patients (aged >15 years) with culture-confirmed melioidosis who had completed 12 weeks of oral TMP-SMX treatment and had no clinical evidence of active melioidosis. Every participant had a chest X-ray, ultrasonogram, or computed tomography scan at enrollment, based on their organ involvement or positive findings during acute melioidosis. Active melioidosis was defined as remaining abnormal signs and symptoms related to initial sites of melioidosis infection, including evidence of active pulmonary infiltration and residual abscesses in any internal organ. The presence of a scar, fibrosis, or calcification in any internal organ was not defined as active melioidosis. We excluded patients if they were infected with *B. pseudomallei* that was resistant to TMP-SMX, if their melioidosis infection was recurrent (defined as having a previous episode of culture-confirmed melioidosis within the past 2 years), if they had a contraindication to TMP-SMX (pregnancy, lactation, aminotransferase >5 times the upper limit of normal, known glucose-6-phosphate dehydrogenase deficiency, or history of hypersensitivity to TMP-SMX), or if they had a history of a grade 3 or 4 adverse drug reaction [17] during their first 12 weeks of TMP-SMX treatment. Minimal inhibitory concentration by E-test was done to confirm TMP-SMX resistance if the result from disc diffusion testing reported TMP-SMX resistance [18, 19].

All centers committed to follow the most up-to-date guidelines for treatment of melioidosis, including a weight-based dosage of TMP-SMX during the oral eradication treatment [16, 20]. In short, if patients had a body weight of <40 kg, the dose used was 160/800 mg of TMP/SMX twice daily; for a body weight of 40–60 kg, the dose was 240/1200 mg of TMP/SMX twice daily; and for a body weight >60 kg, the dose was 320/1600 mg of TMP/SMX twice daily. The dose was adjusted according to the glomerular filtration rate in patients with renal impairment [16, 20].

The methods and results are reported according to the CONSORT (Consolidated Standards of Reporting Trials) statement for noninferiority trials (Supplementary File 1). The study protocol was approved by the local ethical committee and the institutional review boards of all participating hospitals. The study was reviewed and monitored by an independent data safety and monitoring board. Written informed consent was obtained from all participants before enrollment. This trial is registered with ClinicalTrials.gov, NCT01420341.

### Randomization and Masking

We randomly allocated participants in a 1:1 ratio to either a 12-week or a 20-week regimen of oral eradication treatment. Randomization codes were computer generated and were stratified by center with the use of random permuted block (block sizes of 10) using an open-access Random Allocation Software program (http://mahmoodsaghaei.tripod.com/Softwares/randalloc.html). Centers were provided with sealed envelopes with tamper-proof closure strips, to be opened in ascending order. Each envelope contained the random regimen allocation. Periodic checks ensured that sites had the correct number of envelopes, that they were intact, and that the sequential numbering system was maintained. Participants and clinicians were all informed of the allocated regimens.

### Procedures

The patients either stopped oral eradication treatment (12-week regimen) or continued treatment for another 8 weeks (20-week regimen). In cases where patients had received TMP-SMX plus doxycycline during the first 12 weeks of treatment, only TMP-SMX was continued for another 8 weeks.

After enrollment, we followed up patients at Week 8 and Months 4, 8, and 12. Patients who did not attend scheduled appointments were contacted by telephone. At each clinical visit, we undertook a clinical assessment. Complete blood count, erythrocyte sedimentation rate, blood sugar, creatinine, electrolyte, and liver function tests were tested at enrollment and at Week 8. Drug compliance was assessed at enrollment and at Week 8 by pill counts and interviews by a study nurse. In the 20-week group, co-trimoxazole was stopped if patients developed severe adverse drug reactions or were unable to tolerate the drug, and the treatment was switched to amoxicillin-clavulanic acid to complete 20 weeks of eradication treatment.

### Outcomes

The primary outcome of the study was culture-confirmed recurrent melioidosis, which was defined as the new clinical presentation of infection, in association with at least 1 culture from any site positive for *B*. *pseudomallei*, within 1 year after enrollment. Secondary outcomes were overall recurrent melioidosis (culture-confirmed recurrent melioidosis plus clinical recurrence), mortality, adverse drug reactions, and drug compliance. Clinical recurrent melioidosis was defined as the development of new symptoms and signs of infection that were consistent with melioidosis, but in the absence of a positive *B*. *pseudomallei* culture from any site. Adverse drug reactions were graded according to the National Cancer Institute Common Toxicity Criteria [17]. The secondary composite end point, combining overall recurrent melioidosis and mortality, was assessed in a post hoc analysis.

### Statistical Analysis

The trial was powered for a noninferiority design because the intervention group (12-week regimen) received a shorter duration of antimicrobial therapy than the control group (20-week regimen). We hypothesized that both regimens had equivalent efficacy. The expected incidence rate for the primary end point was based on the results of the most recent previous RCT [16]. The noninferiority margin was defined by the study steering committee as a hazard ratio (HR) for culture-confirmed recurrent melioidosis of 2.0. We calculated that 800 participants were needed to determine noninferiority with a power of 80% at an alpha error of 5%. The trial recruitment was terminated at 667 participants after consulting with the data safety monitoring board at Year 7 of the study (June 2018), due to futility of the primary outcome. The loss of power was mitigated using a secondary composite end point combining overall recurrence and mortality. The noninferiority margin defined was used for both the primary and secondary end points.

All patients were analyzed on the basis of intention to treat. Continuous variables are presented as medians and interquartile ranges. Categorical data are presented as numbers (%). We did survival analyses using the Kaplan-Meier method and Cox proportional hazards models. Time was measured from the day of study enrollment. We calculated the probabilities of event outcomes at each time point using the Kaplan-Meier method. For the primary analysis with culture-confirmed recurrent melioidosis as the failure outcome, participants were censored on the day of the last follow-up or death due to other causes. We tested noninferiority of the 12-week regimen by calculating the HR for the efficacy of the 12-week regimen over that of the 20-week regimen, and compared the upper limit of the 95% confidence interval (CI) to the noninferiority margin. To accept the noninferiority margin of the 12-week regimen, the upper limit of the 95% CI needed to be equal to or less than 2.0. A 1-sided test at an alpha error of 2.5% was also calculated for the noninferiority margin.

For the safety analyses, we assessed the proportions of patients by regimen group who developed adverse drug reactions at Week 8 after enrollment. We compared proportions using the Fisher’s exact test and continuous variables using the Mann-Whitney test. We also conducted a sensitivity analysis by using univariable and multivariable Cox proportional hazards models for primary and secondary end points. Interaction tests were planned to evaluate whether effectiveness of the treatment regimens was different among patients with localized melioidosis or blood cultures positive for *B*. *pseudomallei*. We used STATA (version 14.2; College Station, Texas) for all statistical analyses.

## RESULTS

**Figure 1.**
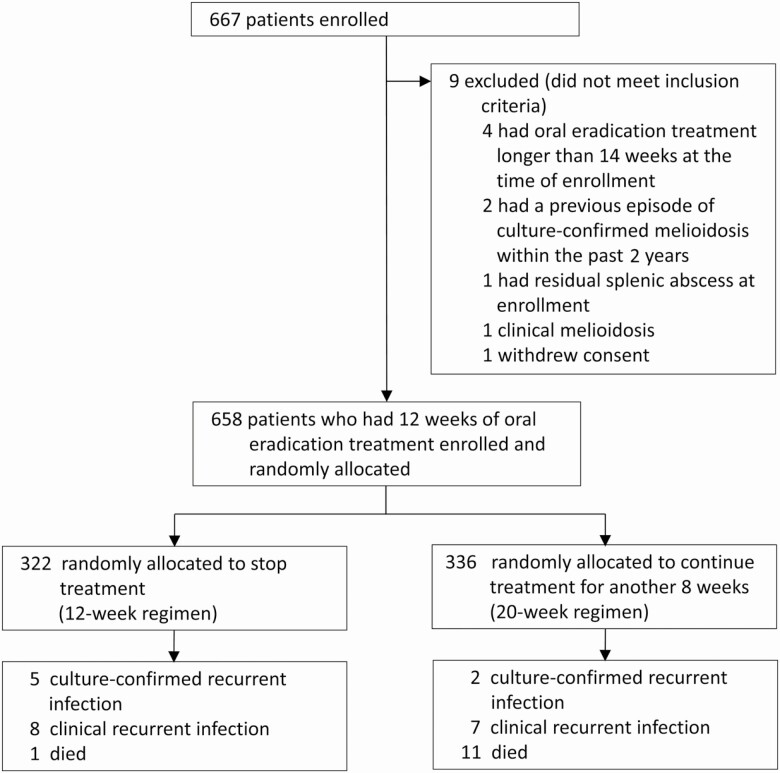
Flow diagram.

Between August 2011 and June 2018, we enrolled 667 patients; 9 patients were found to be ineligible (Figure 1). Of 658 patients who had TMP-SMX as oral eradication treatment for 12 weeks and had no clinical evidence of active melioidosis, 322 (49%) were randomly assigned to stop the treatment (12-week regimen) and 336 (51%) were randomized to continue the treatment for another 8 weeks (20-week regimen). Baseline characteristics were comparable between the 2 groups (Table 1). There were no missing data for baseline characteristics. Overall, 30 patients (5%) did not require parenteral antimicrobial treatment, and 366 (56%) were deemed to require longer than 14 days of parenteral therapy before starting oral treatment. Most patients had a bodyweight of 40–60 kg and received 240/1200 mg of TMP/SMX twice daily. During their first 12 weeks of oral eradication treatment prior to enrollment, 615 (93%) patients received TMP-SMX alone, while 43 (7%) patients received TMP-SMX plus doxycycline.

**Table 1.** Baseline Characteristics

Characteristics	12-Week Group, n = 322	20-Week Group, n = 336
Study site		
Sunpasitthiprasong Hospital, Ubon Ratchathani	161 (50)	164 (49)
Srinagarind Hospital, Khon Kaen	42 (13)	43 (13)
Khon Kaen Hospital, Khon Kaen	15 (5)	18 (5)
Nakhonphanom Hospital, Nakhonphanom	47 (15)	52 (15)
Udon Thani Hospital, Udon Thani	29 (9)	30 (9)
Srisaket Hospital, Srisaket	13 (4)	13 (4)
Chaiyaphum Hospital, Chaiyaphum	12 (4)	13 (4)
Maharat Nakhonratchasima Hospital, Nakhonratchasima	3 (1)	3 (1)
Sex, men	212 (66)	228 (68)
Age, years	54 (45–63)	54 (44–62)
Underlying diseases		
Diabetes mellitus	231 (72)	233 (69)
Chronic kidney disease or renal stones	52 (16)	37 (11)
Chronic liver disease	13 (4)	21 (6)
Thalassemia	5 (2)	12 (4)
Other diseases^a^	33 (10)	30 (9)
Distribution of melioidosis^b^		
Localized	122 (38)	110 (33)
Multifocal	22 (7)	17 (5)
Bacteremic	130 (40)	154 (46)
Disseminated	48 (15)	55 (16)
Organ involvement^c^		
Pneumonia	118 (37)	115 (34)
Skin or subcutaneous abscess	74 (23)	66 (20)
Splenic abscess	42 (13)	44 (13)
Liver abscess	30 (9)	44 (13)
Arthritis	33 (10)	27 (8)
Urinary tract infection	17 (5)	30 (9)
Lymphadenopathy	11 (3)	5 (1)
Other^d^	13 (4)	16 (5)
Duration of parenteral antimicrobials before the start of oral eradication treatment		
None	18 (6)	12 (4)
1–14 days	119 (37)	143 (43)
15–28 days	156 (48)	148 (44)
>29 days	29 (9)	33 (10)
Regimen of oral eradication treatment during the first 12 weeks of oral eradication treatment		
TMP-SMX	302 (94)	313 (93)
TMP-SMX plus doxycycline	20 (6)	23 (7)
Dosage of TMP-SMX received		
160/800 mg twice daily	7 (2)	11 (3)
240/1200 mg twice daily	202 (63)	192 (57)
320/1600 mg twice daily	113 (35)	133 (40)

Data are shown as n (%) or median (interquartile range).

Abbreviations: HIV, human immunodeficiency virus; TMP-SMX, trimethoprim-sulfamethoxazole.

^a^Included steroid intake (19), chronic obstructive pulmonary disease (13), cancer (10), gouty arthritis (10), ischemic heart disease (9), connective tissue diseases (8) and immunosuppressive drug intake (4), and HIV infection (3)

^b^Localized was defined as a single focus of infection and a negative blood culture result; multifocal as >1 noncontiguous focus of infection and a negative blood culture result; bacteremic as a positive blood culture result plus a single or no identifiable focus of infection; and disseminated as a positive blood culture result plus >1 noncontiguous focus of infection

^c^Organ involvement was defined as the presence of clinical features and/or clinical specimen taken from the organ that was culture positive for *Burkholderia pseudomallei*

^d^Included pleuritis or pleural effusion (11), mycotic aneurysm (6), prostatic abscess (3), sinusitis (3), central nervous system infection (2), peritonitis (2), pericarditis (1), and testicular abscess (1)

There was at least 1 follow-up assessment for 650 of the patients (99%). At Week 8 after enrollment, 301 (93%) patients in the 12-week group and 321 (96%) in the 20-week group came for the follow-up, which included a clinical evaluation and laboratory tests (*P* = .25). The 1-year follow-up period was completed by 275 patients (85%) in the 12-week group and 293 (87%) in the 20-week group (*P* = .58).

**Figure 2.**
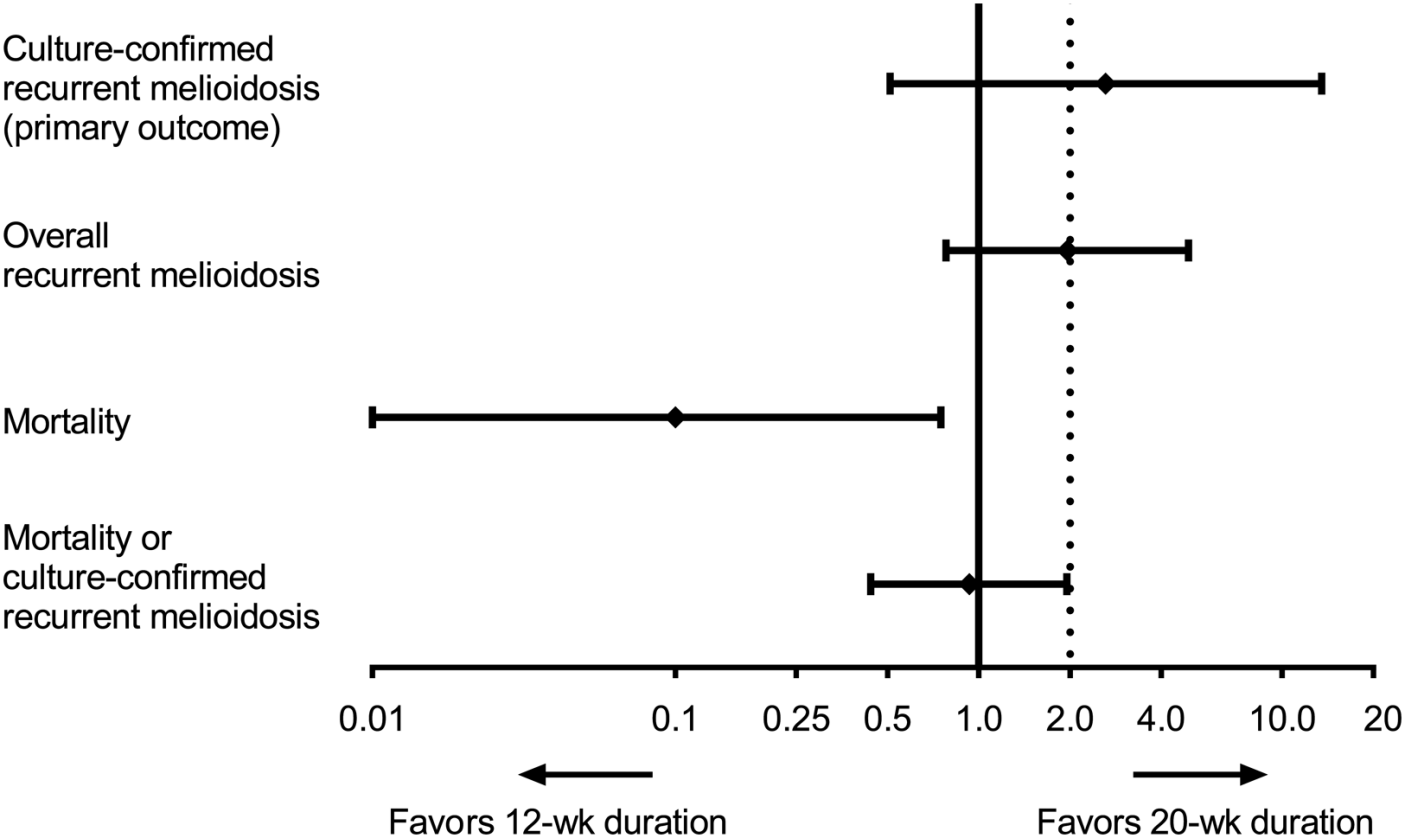
Non-inferiority of 12-week regimen relative to 20-week regimen. Data points are the point estimate of the HR between the 12-week regimen and 20-week regimen. Error bars are 95% CIs. Clinical equivalence of 12-week regimen would be accepted if the upper bound of the 95% CI of the HR for culture-confirmed recurrent melioidosis (primary end point) was below the predefined noninferiority margin (HR 2.0; dotted line). Abbreviations: CI, confidence interval; HR, hazard ratio.

There were 5 patients (2%) in the 12-week group and 2 patients (1%) in the 20-week group who developed culture-confirmed recurrent melioidosis (HR, 2.66; 95% CI, .52–13.7; *P* = .24; Table 2). The criterion for noninferiority of the primary event was not met, because the upper bound of the 95% CI was higher than the predefined noninferiority margin (1-sided *P* = .37; Figure 2). The probability of having culture-confirmed recurrent melioidosis within 1 year of enrollment was 1%. Of 7 culture-confirmed melioidosis cases, 1 (in the 12-week group) occurred during the first 8 weeks of follow-up, and 6 occurred between Week 8 and Year 1 after enrollment.

**Table 2.** Outcomes of the Study

	12-Week Group, n = 322	20-Week Group, n = 336	HR (95% CI)
Recurrent melioidosis			
Culture-confirmed	5 (2)	2 (1)	2.66 (.52–13.69)
Clinical	8 (2)	5 (1)	
Overall	13 (4)	7 (2)	1.99 (.79–4.98)
Mortality			
Due to recurrent melioidosis	1 (.3)	3 (1)	
Due to other causes^a^	0 (0)	8 (2)	
Overall	1 (.3)	11 (3)	.10 (.01–.74)
Recurrent melioidosis or mortality			
Overall	13 (4)	15 (4)	.93 (.44–1.96)

Data are shown as n (%).

Abbreviations: CI confidence interval; HR, hazard ratio.

^a^Included *Klebsiella pneumoniae* bacteremia (1), pneumonia plus extended-spectrum beta-lactamase–producing *Escherichia coli* bacteremia (1), *Aspergillus* endocarditis plus *Enterococcus* spp. bacteremia (1), liver hepatoma (1), hypopharyngeal cancer (1), acute myocardial infarction (1), chronic heart disease with cardiogenic shock (1), and end-stage renal disease with pulmonary edema and cardiogenic shock (1).

**Figure 3.**
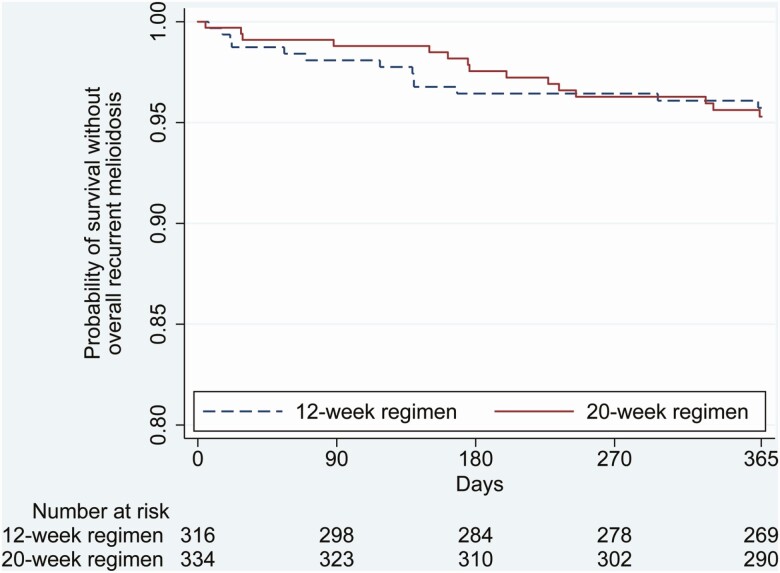
Kaplan-Meier curves of probability without overall recurrent melioidosis and mortality.

A comparison of secondary end points in the 2 arms is shown in Table 2. We observed no difference for overall recurrent melioidosis (*P* = .14). However, overall mortality was lower in the 12-week group (0.3%; 1/322), compared with the 20-week group (3%; 11/336; HR, 0.10; 95% CI, .01–.74; *P* = .03). The causes of death were culture-confirmed recurrent melioidosis (1 patient), clinical recurrent melioidosis (3 patients), and other causes (8 patients). No deaths were attributed to an adverse reaction to TMP-SMX. A composite secondary end point combining overall recurrent melioidosis and mortality was not different between the 2 groups (HR, 0.93; 95% CI, .44–1.96; *P* = .85; Figure 3). The criterion for noninferiority was met because the upper bound of the 95% CI was lower than the predefined noninferiority margin (1-sided *P* = .022; Figure 2).

The proportion of patients having adverse events at Week 8 after enrollment was lower in the 12-week group than in the 20-week group (7% vs 13%, respectively; *P* = .01; Supplementary Table 1). Of the 20-week group, all 321 patients who presented at Week 8 follow-up reported completion of their treatment. None required switching to the second-line drug regimen. Common adverse events were anemia, hyponatremia, vomiting, and hyperkalemia. Serious adverse events (grade 4) were found in 2 patients; these serious adverse events were severe hypokalemia (1 in 20-week group) and severe hyponatremia (1 in 12-week group).

In a sensitivity analysis, we developed univariable and multivariable Cox proportional hazard models to explore whether any factors or interactions were associated with the end points. We found that no factors were associated with culture-confirmed recurrent melioidosis (Supplementary Table 2). Using a multivariable Cox model, male gender and a longer duration of parenteral antimicrobials before starting the oral eradication treatment were associated with overall recurrent melioidosis or death (Supplementary Table 3). None of the interaction tests were positive.

## DISCUSSION

Our findings support the recommendation for 12 weeks of oral eradication treatment for melioidosis. In the primary analysis for culture-confirmed recurrent melioidosis, our study did not show that the 12-week regimen was noninferior to the 20-week regimen. However, our suggestion is derived from the findings that the 12-week regimen was associated with lower total mortality, and that the 12-week regimen was not inferior to 20-week regimen for the secondary composite end point, combining overall recurrent melioidosis and mortality.

The probability of having culture-confirmed recurrent melioidosis within 1 year of enrollment in our study (1%) was lower than that seen in a previous RCT (3%) [16]. The previous study enrolled patients at the start of oral eradication treatment [16], where 18% of the enrolled patients did not complete 12 weeks of treatment, and 5% of patients had to extend treatment beyond 20 weeks due to clinical evidence of residual infection. In this study, only patients who had already had 12 weeks of TMP-SMX treatment and had no clinical evidence of residual infection were enrolled. Moreover, no patients in this study needed to switch to the second-line drug regimen, while 15% of patients in the previous trial needed to switch treatment to amoxicillin-clavulanic acid because of adverse drug reactions [16]. Switches to the second-line drug regimens normally occur during the first 12 weeks of treatment due to adverse drug events. Poor drug adherence, requiring longer than 20 weeks of treatment and switching to second-line drug regimens, are shown to be associated with a higher risk of recurrent melioidosis in a previous retrospective study [8].

We derived our suggestion based on the thought processes recommended when the primary outcome is negative [21]. As we observed the lower total mortality and noninferiority of the secondary composite end point in the 12-week regimen group, we considered that our study should be interpreted constructively. We consider that the primary end point defined is appropriate. This is because the main objective of oral eradication treatment is to reduce the risk of recrudescence or relapse of the primary infection, which commonly occurs during the first year after the completion of initial parenteral antimicrobial treatment [8, 9]. Although there is a concern that the 12-week regimen may be associated with a higher risk of recurrent melioidosis, the risk is not observed in our study. To the best of our knowledge, this study is the largest RCT of oral eradication treatment [12–16]. We carefully considered whether findings of secondary outcomes could be used to support 1 recommendation (12-week regimen in Australia) over the other (20-week regimen in Thailand). The lower overall mortality in the 12-week regimen could be due to chance. This is because many of the causes of mortality in the 20-week regimen group are noncommunicable diseases and infections caused by other organisms. Longer and unnecessary exposure to TMP-SMX might reduce liver and kidney functions of patients, as shown by higher adverse events at the Week 8 follow-up, but this is unlikely to be a direct explanation for the higher mortality. The secondary composite end point could increase the power of the study, and provide some additional evidence [21]. The noninferiority of the secondary composite end point is also supported by preexisting evidence in Australia, showing that 12 weeks of TMP-SMX alone is adequate [10, 11], and in Thailand [8], showing that risk of relapse is not significantly different if the treatment is longer than 8 weeks.

Our trial was intentionally designed to be a pragmatic RCT [22]. First, we minimized the exclusion criteria. Second, we enrolled patients with heterogenous clinical presentation of acute melioidosis and underlying diseases. Our main inclusion criterion was having no clinical evidence of a residual infection after 12 weeks of oral TMP-SMX treatment. Third, no placebo was used: the randomization was between stopping and continuing treatment for another 8 weeks. Fourth, both patients and clinicians were aware of the allocation. Fifth, we assessed the effectiveness of available medicine and regimens [22]. We considered that a typical double-blinded placebo-controlled study design, in which patients would be enrolled at the start of oral treatment and a placebo/active drug would be introduced for Weeks 12 to 20 of a 20-week regimen, would not be practical in our settings and would not test the effectiveness of stopping at 12 weeks (many patients would be unable to stop oral eradication treatment at 12 weeks). During the trial, 5 patients died of noncommunicable diseases, reflecting the real-life nature of the study [22].

Our trial has some limitations. We did not exclude patients with central nervous system (CNS) infections. Both patients with a CNS infection (1 in each treatment arm) survived without recurrent melioidosis at 1 year after enrollment. The use of a 12-week TMP-SMX regimen in patients with CNS infection may not be generalizable, as the sample size of patients with CNS infection is small in our study. Our trial was restricted to patients who had no clinical evidence of active melioidosis after oral eradication treatment with TMP-SMX for 12 weeks. Therefore, those who require second-line drug regimens or still have clinical evidence of a residual infection may still need a longer duration of treatment. We did not perform genotyping and could not exclude patients with reinfections.

In conclusion, the findings of our trial support 12 weeks of TMP-SMX as the standard for oral treatment for melioidosis.

## Supplementary Data

Supplementary materials are available at *Clinical Infectious Diseases* online. Consisting of data provided by the authors to benefit the reader, the posted materials are not copyedited and are the sole responsibility of the authors, so questions or comments should be addressed to the corresponding author.

ciaa1084_suppl_Supplementary-File-1

ciaa1084_suppl_Supplementary-Table

## Notes

***Acknowledgments.*** The authors thank all the patients, their relatives, the staff of the hospitals who participated in the study, and the Data and Safety Monitoring Board, including Professors Bart Currie, Boonmee Sathapatayavongs, and Yupin Suputtamongkol.

***Financial support.*** This work was supported by the Higher Education Research Promotion and National Research University Project of Thailand, Office of the Higher Education Commission, through the Center of Excellence in Specific Health Problems in Greater Mekong Sub-region cluster and the Melioidosis Research Center, Khon Kaen University (project number 542040) and by the Wellcome Trust (grant number 106698/B/14/Z).

***Potential conflicts of interest.*** The authors: No reported conflicts of interest. All authors have submitted the ICMJE Form for Disclosure of Potential Conflicts of Interest.
