# Supplementary material for: A Comparison Between 12 Versus 20 Weeks of Trimethoprim-sulfamethoxazole as Oral Eradication Treatment for Melioidosis: An Open-label, Pragmatic, Multicenter, Non-inferiority, Randomized Controlled Trial
Source: Clin Infect Dis. 2020 Jul 29;73(11):e3627–33. doi: 10.1093/cid/ciaa1084 (PMC8662794; doi:10.1093/cid/ciaa1084)
Supplement: ciaa1084_suppl_Supplementary-Table [file ciaa1084_suppl_supplementary-table.docx]

**Supplementary Table 1. Adverse Events at 8 Weeks after Enrollment**

|  | 12-week group  (n=301)^a^ | 20-week group  (n=321)^a^ |
| --- | --- | --- |
| Allergic reactions |  |  |
| Rash | 0 (0) | 3 (1) |
| Gastrointestinal disorders |  |  |
| Elevation of AST/ALT level (>5xULN) | 0 (0) | 1 (0.3) |
| Nausea | 1 (0.3) | 1 (0.3) |
| Vomiting | 4 (1) | 9 (3) |
| Genitourinary disorder |  |  |
| Elevation of creatinine level (>0.5 mg/dl) | 3 (1) | 1 (0.3) |
| Hematologic disorder |  |  |
| Anemia (hemoglobin level decreased >2 g/dl) | 4 (1) | 12 (4) |
| Thrombocytopenia (<50,000 /mm^3^) | 6 (2) | 3 (1) |
| Metabolic disorder |  |  |
| Hyponatremia (<130 mEq/L) | 5 (2) | 11 (3) |
| Hyperkalemia (>6 mEq/L) | 1 (0.3) | 10 (3) |
| Hypokalemia (<3 mEq/L) | 3 (1) | 2 (1) |
| Overall | 23 (7) | 45 (13) |

Data are n (%).

Abbreviations: ALT, alanine aminotransferase; AST, aspartate aminotransferase; ULN, upper limit of normal.

^a^301 (93%) patients in the 12-week regimen group and 321 (96%) in the 20-week regimen group came for the follow-up at weeks 8 for clinical evaluation and laboratory tests for adverse events.

**Supplementary Table 2. Factors Associated with Culture-confirmed Recurrent Melioidosis**

| Factors | HR (95% CI)^a^ | *P*Value |
| --- | --- | --- |
| 12-week regimen group | 2.66 (0.52-13.7) | .24 |
| Sex, men | 3.12 (0.37-26.1) | .29 |
| Age, years | 1.02 (0.96-1.09) | .46 |
| Diabetes mellitus | 1.16 (0.21-6.11) | .87 |
| Localized distribution of melioidosis | 0.63 (0.12-3.29) | .59 |
| Blood culture positive | 2.15 (0.41-11.3) | .36 |
| Duration of parenteral antimicrobials before starting the oral eradication treatment (days) | 1.01 (0.93-1.09) | .83 |

Abbreviations: HR, hazard ratio; CI, confidence interval.

^a^HR was estimated by univariable Cox proportional hazard model stratified by center

**Supplementary Table 3. Factors Associated with Overall Recurrent Melioidosis or Mortality**

| Factors | HR (95% CI)^a^ | *P*Value | Adjusted HR (95% CI) | *P*Value |
| --- | --- | --- | --- | --- |
| 12-week regimen group | 0.93 (0.44-1.96) | .85 | 1.00 (0.47-2.11) | > .99 |
| Sex, men | 2.95 (1.02-8.55) | .05 | 3.01 (1.04-8.71) | .04 |
| Age, years | 1.02 (0.99-1.05) | .15 | - | - |
| Diabetes mellitus | 0.59 (0.27-1.26) | .17 | - | - |
| Localized distribution of melioidosis | 0.63 (0.28-1.45) | .28 | - | - |
| Blood culture positive | 1.50 (0.69-3.29) | .31 | - | - |
| Duration of parenteral antimicrobials before starting the oral eradication treatment (days) | 1.03 (1.00-1.07) | .05 | 1.03 (1.00-1.07) | .04 |

Abbreviations: HR, hazard ratio; CI, confidence interval.

^a^HR was estimated by univariable Cox proportional hazard model stratified by center
